# Exploring Sources, Biological Functions, and Potential Applications of the Ubiquitous Marine Cyclic Dipeptide: A Concise Review of Cyclic Glycine-Proline

**DOI:** 10.3390/md22060271

**Published:** 2024-06-12

**Authors:** Lei Hu, Jing Lin, Fei Qin, Li Xu, Lianzhong Luo

**Affiliations:** 1 Xiamen Key Laboratory of Marine Medicinal Natural Product Resources, Xiamen Medical College, Xiamen 361023, China; L13959531069@163.com (J.L.); 201500010325@xmmc.edu.cn (F.Q.); xli@xmmc.edu.cn (L.X.); 2 Fujian Province University Marine Biopharmaceutical Resource Engineering Research Center, Xiamen Medical College, Xiamen 361023, China; 3 School of Pharmacy and Pharmaceutical Sciences, Xiamen Medical College, Xiamen 361023, China

**Keywords:** marine cyclic dipeptide, marine origins, endogenous, insulin-like growth factor-1 homeostasis, building block, drug discovery

## Abstract

Cyclic glycine-proline (**cGP**), a prevalent marine cyclic dipeptide, possesses a distinct pyrrolidine-2,5-dione scaffold, which contributes to the chemical diversity and broad bioactivities of **cGP**. The diverse sources from marine-related, endogenous biological, and synthetic pathways and the in vitro and in vivo activities of **cGP** are reviewed. The potential applications for **cGP** are also explored. In particular, the pivotal roles of **cGP** in regulating insulin-like growth factor-1 homeostasis, enhancing neuroprotective effects, and improving neurotrophic function in central nervous system diseases are described. The potential roles of this endogenous cyclic peptide in drug development and healthcare initiatives are also highlighted. This review underscores the significance of **cGP** as a fundamental building block in drug discovery with exceptional drug-like properties and safety. By elucidating the considerable value of **cGP**, this review aims to reignite interest in **cGP**-related research within marine medicinal chemistry and synthetic biology.

## 1. Introduction

Recently, the concept of ‘drugs from the sea’ has gained widespread acceptance, exemplified by marine-derived cyclic peptides like ziconotide and plitidepsin, which have been approved for the treatment of severe pain and multiple myeloma, respectively [1,2,3]. Interest in marine-derived cyclic peptides in drug discovery surged from 2010 to 2020; an average of 268 related publications per year described the broad biological activity and structural diversity of cyclic peptides [4]. Cyclic peptides lack the inherent disadvantages of linear peptides, including poor membrane permeability and enzymatic stability [5]. Their constrained conformation results in minimal entropic cost when binding to biological targets. Thus, cyclic peptides exhibit superior pharmacological properties compared to their linear counterparts [6]. The unique characteristics of cyclic peptides foster the development of novel drugs with extensive prospects, including antibiotics, immunosuppressants, and inhibitors of protein–protein interaction [7].

**Figure 1:**
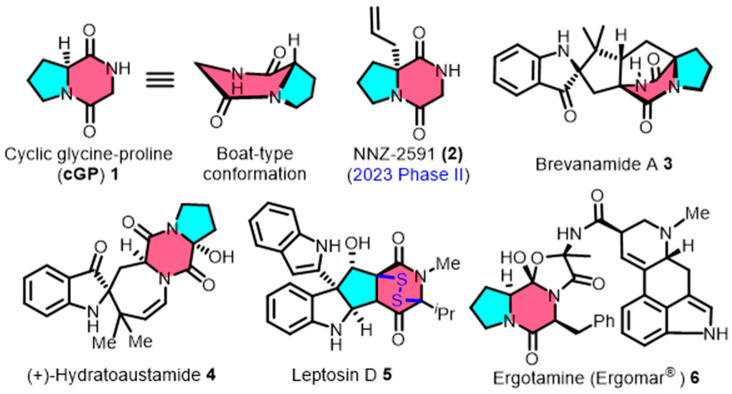
The spatial conformation of cyclic glycine-proline (**cGP**), along with representative clinical drugs and complex natural products containing **cGP** scaffold.

Cyclic glycine-proline (**cGP**, **1**) is a small-molecule cyclic dipeptide, which is widely sourced from the secondary metabolism of marine microorganisms [8,9]. Cyclic GP consists of glycine and proline, which form a distinctive pyrrolidinedione framework and display a unique non-planar stereo structure (Figure 1). This molecule is found in the structural composition of clinical drugs and the fundamental skeletons of complex natural products. For example, the derivative NNZ-2591 (**2**) is currently undergoing Phase II clinical trials for the treatment of developmental neurological disorders [10]. The migraine medication ergotamine (Ergomar^®^) (**6**) also contains **cGP** [11]. Additionally, the **cGP** structure is present in natural products, including brevanamide A (**3**) [12], (+) hydratoaustamide (**4**) [13,14], and leptosin D (**5**) [15]. The cage-shaped bicyclo[2.2.2]diazaoctane (**3**) and the disulfide-bridged epipolythiodioxopiperazines (**5**) both incorporate **cGP** in their construction.

Given the structural importance of **cGP** in drug discovery and its abundance in complex natural products, the potential of **cGP** has been extensively explored. Previous research focused on the optical chirality [16], diverse derivatives, and biological activities of **cGP** [8,9]. However, the potential of **cGP**-containing marine microbial organisms as a promising sustainable drug source has received less attention. This brief review covers various sources of **cGP**, including marine origins, biotransformation, and chemical synthesis, focusing on literature published between 2000 and 2022. Investigations into the in vitro and in vivo activity of **cGP** and the applications of **cGP** in preclinical drug discovery are highlighted. Additionally, future perspectives on engineered biosynthesis derived from marine microbial, diversity-building block libraries, and preclinical neurological drug candidates are discussed. This review provides valuable insights for further research and development of **cGP**.

## 2. Sources: Marine Origins, Endogenous Biological Transformation, and Chemical Synthesis

### 2.1. Marine Origins

The prevalent marine secondary metabolite of **cGP** was first isolated from Gulf of Mexico starfish, and its structure was later confirmed through X-ray analysis by Von Dreele in 1975 [17]. A decade later, the secondary metabolite of **cGP** was also found in *Diodon novemacultus* from the South China Sea [18]. Traces of the cyclic dipeptide have been discovered in various sources, including terrestrial fungi [19,20,21,22,23], lake cyanobacteria [24], animals [25], and even in food items such as coffee [26,27], beef [28], and blackcurrant [29]. However, marine organisms have emerged as a noteworthy natural source of **cGP**. This review primarily focuses on marine sources of **cGP** up to 2022, due to the prevalence of **cGP** in marine natural product chemistry (Table 1) [30,31,32,33,34,35,36,37,38,39,40,41,42,43,44,45,46]. Interestingly, the majority of collection sites for marine sources of **cGP** are located in tropical and subtropical coastal areas [47], and several sites are closely associated with mangroves. In these environments, microorganisms, such as *Penicillium*, *Aspergillus*, and *Streptomyces*, can produce **cGP** and its diverse derivatives. Although extensive research has focused on the structural diversity of this cyclic peptide in marine natural product chemistry, biosynthesis of **cGP** has received less attention. Exploring the biosynthesis of **cGP** using genetically informative microbes may provide a sustainable drug source and offer a novel approach to achieving cyclic peptide green synthesis.

### 2.2. Endogenous Biological Transformation

**Figure 2:**
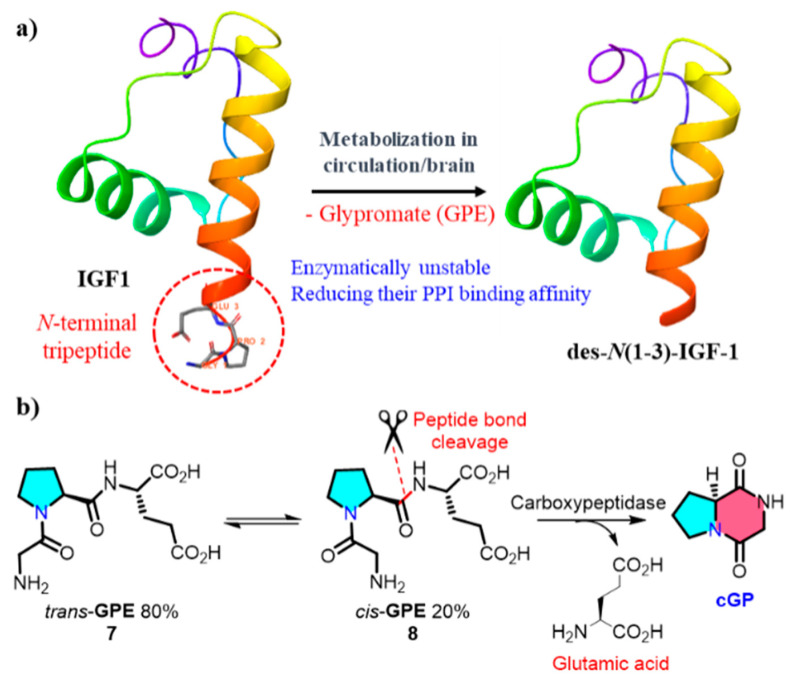
(**a**) Metabolism of IGF-1 within the organism; (**b**) formation of endogenous cyclic glycine-proline (PPI, protein–protein interaction).

An endogenous biological source of **cGP** was identified by Gudasheva et al. in 1996; **cGP** was identified as an endogenous bioactive cyclic dipeptide in the rat brain at 2.8 nmol/g. The anti-amnesic activity of **cGP**, which is associated with memory regulation, was also demonstrated in this study [48]. Insulin-like growth factor-1 (IGF-1) is an insulin-like hormone that plays crucial regulatory roles in human growth, development, and metabolic processes [49]. Clinical trials demonstrated that low concentrations of **cGP** and decreased **cGP**/IGF-1 ratios in the plasma are associated with impaired IGF-1 function during the early stages of stroke. After ischemic brain injury, **cGP** exhibits neuroprotective effects and shares pharmacological similarities with IGF-1 [50,51]. IGF-1 undergoes enzymatic cleavage in the plasma and brain tissues, releasing *des-N*(1-3)-IGF-1 residues and linear glycine-proline glutamic acid (GPE, *cis*/*trans* = 1/4) (Figure 2). The reversible *cis*-GPE (**8**), metabolised by carboxypeptidase to release glutamic acid and **cGP**, dynamically regulates the bioavailability of **cGP** in the circulation to restore the normalisation of IGF-1 [52]. More details on the physiological functions of **cGP** will be elucidated in the next section.

### 2.3. Chemical Synthesis

Hayasaka et al. developed a cost-effective strategy for generating tripeptide components rich in Gly-Pro-Y sequences, which were derived from the enzymatic hydrolysis of proteins. Subsequent heating converts the tripeptide into the cyclic glycine-proline dipeptide, with a conversion rate of 78%. This method represents an economically viable biotransformation protocol for achieving large-scale synthesis [53].

**Figure 3:**
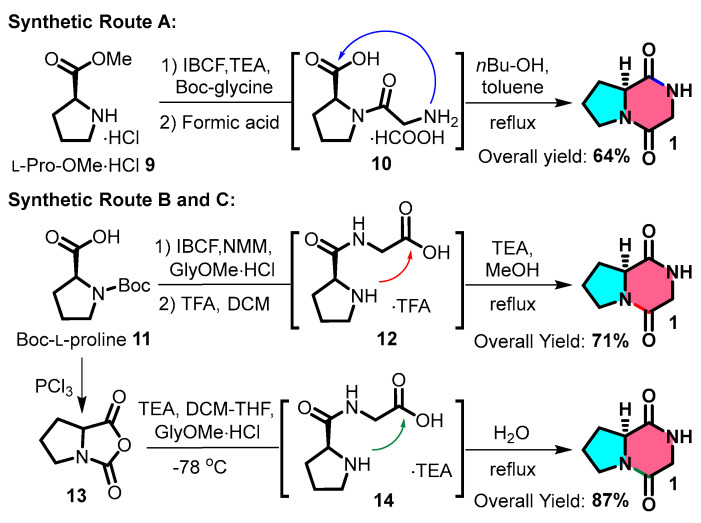
General synthetic route for cyclic glycine-proline.

The routine synthesis of **cGP** typically involves several steps. *N*-Boc-protected glycine can be coupled with methylated _L_-proline **9** using isobutyl chloroformate and triethylamine (TEA). After removing the *N*-Boc protecting group, the intermediate undergoes intramolecular cyclisation upon heating, producing a cyclic dipeptide at a 64% yield (Figure 3: Route A). Another method involves a C-N bond-forming sequence using *N*-Boc-_L_-proline **11**, followed by the steps listed above to produce a cyclic dipeptide at a 71% yield (Figure 3: Route B) [16,54,55]. A third approach includes acylating *N*-Boc-_L_-proline and coupling the resulting product with glycine methyl ester hydrochloride; refluxing with water produces a cyclic dipeptide at a yield of 87% (Figure 3: Route C) [56]. The third approach (synthetic pathway C) exhibits superior atom economy; peptide bond formation is achieved without condensing agents, providing a potential strategy for streamlined, multistep continuous flow synthesis in the future.

**Figure 4:**
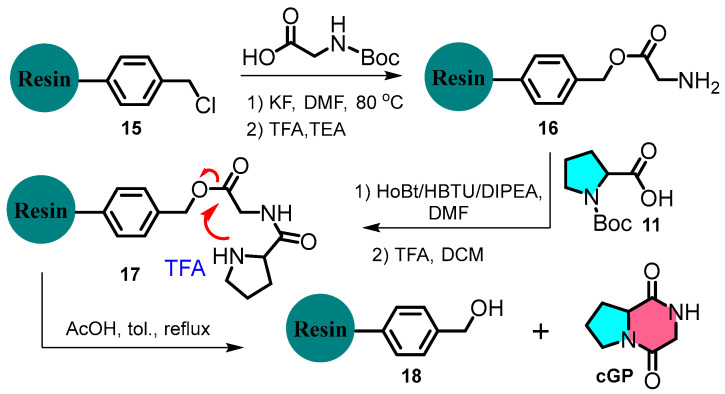
Solid-phase peptide synthesis of cyclic glycine-proline.

Chen et al. devised a rapid and flexible method to expand cyclic dipeptide diversity using immobilised Merrifield solid-phase peptide synthesis (Figure 4) [28]. For example, the *N*-Boc-glycine residue undergoes deprotection and condensation with *N*-Boc-_L_-proline, catalysed by HBTU/HOBt. The product is subsequently treated with TFA and TEA to form a cyclic dipeptide. This strategy contrasts with liquid-phase peptide synthesis and offers an efficient, versatile, and scalable approach to cyclic dipeptide synthesis.

**Figure 5:**
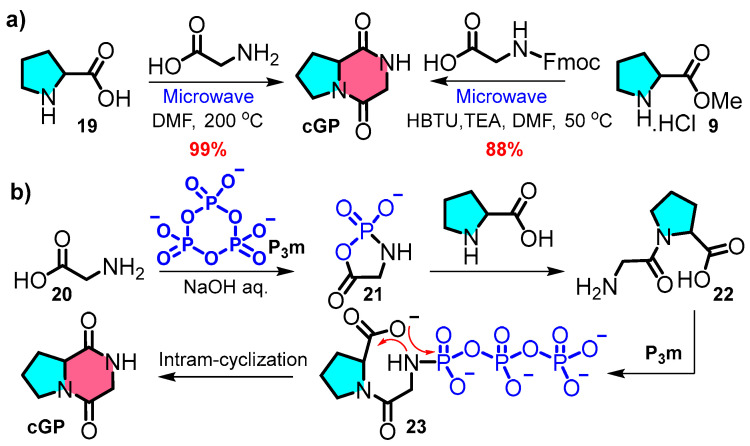
(**a**) One-pot microwave protocol; (**b**) biomimetic synthetic route.

Compared to the traditional multistep synthesis, a concise and efficient synthetic strategy offers a novel approach aligned with the principles of atom economy and environmentally friendly synthesis. Ahonen et al. introduced an effective green synthetic one-step synthesis protocol at a 100-milligram scale based on microwave reactions (Figure 5a left) [57]. The interaction of _L_-proline with glycine under high-temperature microwave conditions leads to rapid product formation through direct cooling and filtration and eliminates the need for chromatography purification. A similar ultrasound-assisted process, incorporating intramolecular cyclisation within a one-pot synthesis, was reported by Poonia et al. (Figure 5a right) [58]. The streamlined synthesis of cyclic peptides from free amino acids saves time and costs and reduces waste.

Zhao et al. demonstrated the prebiotic synthesis of cyclic peptides. In this reaction, the spontaneous formation of cyclic peptides from linear dipeptide **22** was facilitated by intramolecular cyclisation and had a calculated reaction barrier of 33.2 kcal·mol−^1^ [59,60]. Furthermore, the phosphorylation of glycine **20** by trimetaphosphate (P_3_m) generates an active five-membered intermediate **21**, which interacts with _L_-proline and is further catalysed by P_3_m to yield **cGP** with a theoretical yield of 97% (Figure 5b).

## 3. In Vitro and In Vivo Effects

In vitro screening in tumour cells revealed the anti-tumour effects of **cGP**, ranging from modest to weak. For example, **cGP** exhibited an IC_50_ value of 101.8 µM against HepG2 cells [42] and an IC_50_ value of 206 µM against A549 cells [23], indicating limited anti-tumour activity. The effects of **cGP** on cytokine release have also been investigated. Jia et al. observed that **cGP**, at a concentration of 5.0 µg/mL, substantially upregulated IFN-c in the murine macrophage-like cell line J774A.1 but had only moderate effects in MCP-1 and IL-10 cells and had only a minor impact on TNF-α secretion [43]. In contrast, Khan et al. detected the inhibition of TNF-α release by **cGP** in LPS-induced RAW 264.7 macrophage, with an IC_50_ value of 4.5 µg/mL [61]. This was accompanied by the downregulation of IL-1β and IL-6 expression. The notable decrease in **cGP** levels during LPS-induced NO production, resulting in minimal cytotoxicity, underscores the promising anti-inflammatory properties of **cGP**. These results emphasise the need for further development of this small cyclic dipeptide as a cytokine modulator.

**Figure 6:**
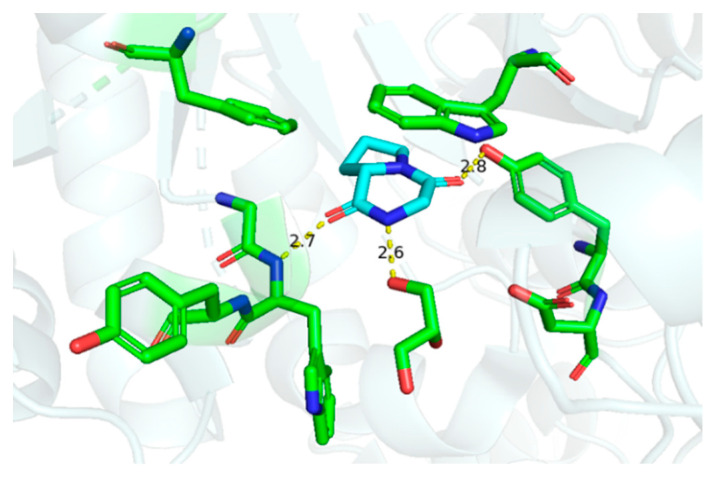
Interactions between cyclic glycine-proline and chitinase (hydrogen bonding distance unit: Å) [62].

Although **cGP** exhibited negligible antimicrobial and antifungal activities against microbes such as *Bacillus subtilis*, *Escherichia coli*, and *Saccharomyces cerevisiae* [34], **cGP** selectively inhibits chitinase enzymes at a concentration of 5.0 mM [62]. Thus, **cGP** is a promising candidate for crop protection and the prevention of nosocomial cross-infection. The crystal structure discloses hydrogen bonding interactions, such as carbonyl and N-H, in the cyclic dipeptide scaffold and amino acid residues, providing a foundation for designing novel inhibitors targeting *Serratia marcescens* chitinase (Figure 6). Moreover, **cGP** markedly enhances antibiotic production in microorganisms, likely due to the stimulation of bacterial quorum-sensing signals [32].

In puncture spray trials, **cGP** exhibited efficient and selective phytotoxicity against the crop weed *A. euphorbiicola* at a concentration of 1.0 mM [63]. Additionally, investigations into its acaricidal activity against *T. urticae* revealed an LC_50_ value of 96 µM [64,65]. Although these screenings yielded moderate results in terms of phytotoxicity and anti-pest activity, **cGP** is a promising candidate for use as a green agricultural product for biological control due to its availability from natural sources and selective inhibitory properties [66].

**Figure 7:**
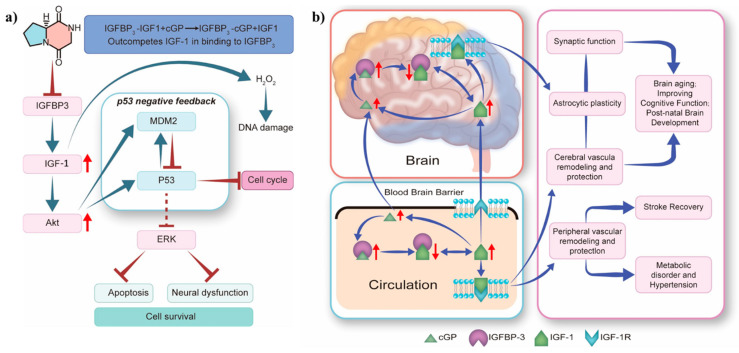
Proposed mechanisms for the effects of cyclic glycine-proline: (**a**) induction of apoptosis in human foetal neural stem cells; (**b**) regulation of IGF-1 homeostasis in the circulatory system and brain tissue. Akt, protein kinase B; **cGP**, cyclic glycine-proline; ERK, extracellular signal-regulated kinase; IGF-1 or IGF1, insulin-like growth factor 1; IGFBP-3 or IGFBP_3_, insulin-like growth factor-binding protein 3; IGF-1R, insulin-like growth factor 1 receptor; MDM2, mouse double minute 2 homologous protein (Note: Redrawn graphics are based on original artwork by Murotomi [67] and Guan [74]. The red arrows on the left indicate the up-regulated levels of related proteins, while the red arrows on the right indicate changes in their relative amounts).

In vitro studies revealed that cyclic dipeptide can alleviate oxidative stress in human foetal neural stem cells (hfNSC). The cyclic dipeptide maintains IGF-1 homeostasis, activates Akt signalling, and enhances MDM2 E3 ubiquitin ligase expression. This regulation influences the interaction between IGF-1 signalling and MDM2-p53 pathways, resulting in a dose-dependent reduction in hfNSC cell death and apoptosis (Figure 7a) [67]. Importantly, **cGP** mimics the pharmacological effects of pramiracetam, including enhanced cognition [48,68], reduced anxiety [69], neuroprotection [51], analgesia [70], and antidepressant properties [71]. Mechanistic studies demonstrate that **cGP** modulates AMPA receptor-mediated currents in Purkinje neuronal cells [72,73]. Furthermore, **cGP** upregulates and activates the AMPA receptor-mediated brain-derived neurotrophic factor-tropomyosin receptor kinase B signalling pathway. These studies highlight the positive allosteric modulator ampakine-like pharmacological characteristics that contribute to the neuropsychotropic effects of **cGP**.

The neuroactive peptide **cGP**, derived from the brain neurotrophic factor IGF-1, regulates IGF-1 homeostasis by modulating the binding of IGF-binding protein 3 (IGFBP-3) to IGF-1. This modulation normalises IGF-1 bioavailability and function, especially in the context of brain ageing and age-related neurological conditions. Additionally, **cGP** maintains IGF-1 function within the optimal physiological range, enhancing function when IGF-1 function is deficient and inhibiting function when IGF-1 is excessive. IGF-1 function is optimised by the inherent affinity for **cGP**, which originates from the binding domain of IGF-1, enabling **cGP** to compete with IGF-1 for IGFBP-3 binding. Thus, IGF-1 function is regulated by the circulating **cGP**/IGF-1 molar ratio. Reduced IGF-1 bioavailability in patients often indicates age-related conditions such as hypertension, stroke, and neurological disorders with cognitive impairment. An increase in the **cGP**/IGF-1 ratio is associated with more favourable clinical outcomes, including improved memory retention and stroke recovery. Moreover, the absorption of **cGP** is enhanced in human tissues, and **cGP** efficiently and directly crosses the blood–brain barrier into the cerebrospinal fluid. Activation of IGF-1 receptors in capillaries promotes astrocyte plasticity and vascular formation to enhance memory (Figure 7b) [74]. According to Fan et al., the intake of exogenous cyclic dipeptide is associated with reduced cardiovascular systolic pressure and improved stroke recovery [29]. For instance, the cognitive health supplement cGPMAX™ Brain Health enhances mental clarity, improves sleep, and balances stress and emotions in older individuals. The outstanding safety profile and beneficial effects of **cGP** on the nervous system emphasise the potential commercial use of **cGP** in the pharmaceutical and healthcare sectors, particularly for the treatment of neurological disorders.

## 4. Exploring Applications for Building Blocks

Structurally, **cGP** consists of lipophilic and hydrophobic pyrrolidine rings with hydrogen bond donors and acceptors. According to Swiss ADME predictions [75,76], **cGP** adheres well to the “Rule of Two” [77,78,79] for drug-likeness building blocks (Table 2). Additionally, **cGP** exhibits a high-saturation carbon atom distribution rate (Fsp_3_) [80] of 0.73, representing remarkable three-dimensional features. Based on the empirical rules from Lipinski [81] and Veber [82], the topological polar surface area (TPSA) [83] of 49.4 Å^2^ for **cGP** exceeds 30.8 Å^2^ (0.2 × MW) [84] and is less than 60–70 Å^2^. Thus, the TPSA falls within the range conducive [85,86] to crossing the blood–brain and intestinal barriers. These predictions are consistent with the exceptional membrane permeability, oral availability, and metabolic stability of **cGP**, which facilitate the optimisation of lead drug-likeness.

**Figure 8:**
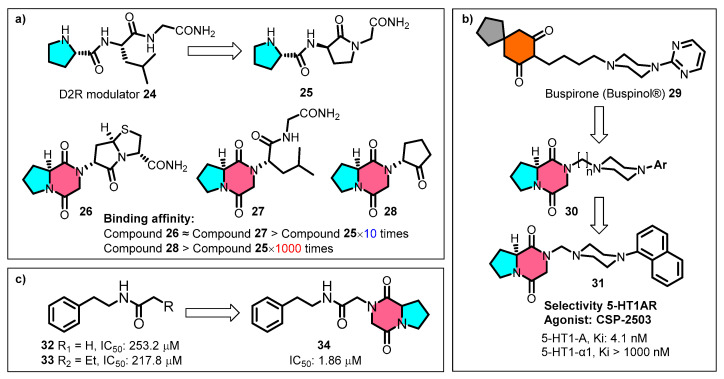
Application of cyclic glycine-proline for building blocks: (**a**) dopamine receptor-regulating peptide (D2R) analogues; (**b**) selective 5-HT1A receptor agonists; (**c**) serotonin-specific receptor (Se-5HTR) inhibitors.

Capitalising on the specific topological molecular structure of **cGP**, Baures et al. replaced the proline portions of the dopamine receptor-regulating peptide **25** with **cGP**, resulting in conformationally restricted analogues **26**–**28** with exponentially improved binding affinities (Figure 8a) [87]. The *N*-positioned pharmacophore cyclopentanone **28** exhibits significantly enhanced receptor affinity by 1000-fold compared to the prototype compound **25**. Lopez-Rodriguez et al. incorporated the cyclic dipeptide as a conformational mimic into the phenylpiperazine pharmacophore scaffold of the anxiolytic agent butorphanol **29**, producing a series of selective 5-HT1A receptor agonists **30** (Figure 8b) [88,89]. The clinical candidate CSP-2503 (**31**) was validated pharmacologically as a 5-HT1AR agonist, acting on both the somatodendritic and postsynaptic sites. CSP-2503 also exhibits high-affinity antagonistic effects on 5-HT2A and 5-HT3 receptors [90,91].

Based on a structure–activity relationship study of *Spodoptera exigua* serotonin-specific receptor (Se-5HTR) inhibitors, Hasan et al. linked the **cGP** fragment to the end of the carbon chain, which enhanced the specificity and competitive inhibitory activity of compound **34** against Se-5HTR (Figure 8c) [92]. This modification resulted in a two-order-of-magnitude improvement in the in vitro immunosuppressive activity compared to the prototype compounds (**32**–**33**). Thus, leveraging the outstanding drug-likeness and bonding affinity advantages of **cGP** provides efficient pharmaceutical building blocks for lead optimisation in the early stages of drug discovery.

## 5. Perspectives and Conclusions

Although the biosynthesis mechanism of **cGP** is largely unexplored, **cGP** may originate from glycine and proline, which are abundant in nature. Peptide biosynthesis involves nonribosomal peptide synthetase (NRPS), encoded by biosynthetic gene clusters (BGCs). NRPS is a core biosynthetic enzyme responsible for the key metabolic step in natural peptide biosynthesis [93,94]. Maiya et al. demonstrated that the biosynthesis pathway of **cGP** compounds involves the BGC ftmA, encoding enzymes for peptide condensation via NRPS to produce the analogue, brevianamide F [95,96]. Further research by Dubois et al. explored the feasibility of constructing the **cGP** framework in natural product biosynthesis using *Escherichia coli*; co-expressing NRPS with a new ribosome-independent enzyme, cyclodipeptide synthase [97,98], results in an increase in the derivative tryprostatin B, yielding up to 26 mg/L [99]. Exploring efficient chemo- and enantio-selective peptidyl transferases from diverse mangrove microbial strains may lay the foundation for the biosynthesis of **cGP** directly from glycine and _L_-proline. Alternatively, integrating BGC profiles from marine microbes with advanced techniques such as CRISPR/Cas9 gene editing, metabolic flux analysis, atmospheric and room temperature plasma mutagenesis, and adaptive laboratory evolution technology may provide insights into the technical challenges associated with extremely low biosynthetic productivity.

Increasing the prevalence of quaternary carbon centres, rather than relying solely on flat aromatic rings, represents a crucial strategy in contemporary drug discovery aimed at transcending two-dimensional molecular structures. This paradigm, often referred to as the ‘escaping the flatland’ [100,101] approach, has been consistently associated with enhanced drug-like properties and the increased success rate of clinical drug candidates. Augmenting the topological polar surface area and introducing three-dimensional spatial characteristics facilitates the optimisation of pharmacological profiles. Consequently, the unique topological scaffold, optical chirality, and chemical diversity inherent to **cGP** render it particularly valuable for early-stage drug discovery applications, including bioisostere and scaffold-hopping endeavours. Nonetheless, the development of precise chiral chemical synthesis methodologies, specifically targeting particular sites to expand building block libraries, remains a challenge. This is particularly true for incorporating new quaternary carbon centres and introducing functional substituents containing fluorine or boron.

NNZ-2591, an allylic analogue situated at the quaternary bridgehead carbon centre, has undergone clinical trials for the treatment of developmental neurological disorders, including Rett syndrome and fragile X syndrome [102,103]. Additionally, a phase II study targeting Prader–Willi syndrome received FDA accelerated approval in 2023 [104]. Given the role of **cGP** in regulating IGF-1 across various neurological and metabolic disorders, exploring the structure–activity relationship of **cGP**-derived compounds could offer valuable insights for the development of preventive healthcare and treatment strategies.

In summary, this review explores the diverse sources of **cGP** from marine-related, endogenous biological, and synthetic pathways, along with its in vitro and in vivo activities. The presence of **cGP** sourced sustainably from marine mangrove microbial origins, coupled with cutting-edge synthetic biological methodologies, has ignited significant interest in its biosynthesis. Moreover, the potential roles of this endogenous cyclic peptide in drug development and healthcare initiatives are emphasised. For example, the pivotal role of **cGP** in regulating insulin-like growth factor-1 homeostasis positively impacts the treatment of central nervous system diseases. Finally, the precise binding affinity, pharmacological attributes, exceptional drug-like characteristics, and safety profile of **cGP** collectively expand its applications as a fundamental building block, paving the way for novel drug discoveries in the early stages.

## Figures and Tables

**Table 1:** Overview of marine sources of cyclic glycine-proline (**cGP**) with their bioactivities from 2000 to 2022.

Sources	Species	Habitats	Bioactivities	Refs
**Bacteria**	*B.coagulans*	Yellow croaker, Zhejiang, China	-- ^a^	[30]
	*Oceanisphaera* sp.	Halong Bay sediment, Vietnam	-- ^a^	[31]
	*Pseudoalteromonas luteoviolacea*	Hawaiian seaweed ^a^	Stimulate bacteria for quorum-sensing signals	[32]
	*Psychrophilic yeast* *Glaciozyma antarctica* PI12	Casey Research Station, Antarctica	Weak cytotoxicity or anti-oxidation	[33]
	*Ruegeria* Strain	Sponge *Suberites domuncula* ^a^	No antimicrobial or antifungal	[34]
**Fungus**	*Penicillium oxalicum*	Coastal intertidal zone, Tsingtao, China	-- ^a^	[35]
	*Ascotricha* sp.	Mudflat soil, Zhejiang, China	Weak cytotoxicity	[36]
	*Penicillium oxalicum*	Mangrove plant root, Hainan, China	No cytotoxicity	[37]
	*Aspergillus terreus* HT-1	Mangrove plant root, Hainan, China	-- ^a^	[38]
	Mangrove endophytic fungus	Mangrove plant seed, Hong Kong, China	-- ^a^	[39]
**Actinomycetes**	*Streptomyces* sp. G261	Sediment, Cham Islands, Vietnam	-- ^a^	[40]
	*Streptomyces fradiae*	Prickly pen shell, Van Phong, Vietnam	-- ^a^	[41]
	*Streptomyces* sp.	Mangrove rhizosphere soil, Fujian, China	HepG2 cells (IC_50_: 101.8 μM)	[42]
**Sponge**	*Callyspongia* sp.	South China Sea	Mild macrophage cytokines stimulator	[43]
	*Callyspongia* sp.	South China Sea	No cytotoxicity	[44]
	*Axinella* sp.	South China Sea	-- ^a^	[45]
	*Tedania* sp.	Zhanjiang, China	-- ^a^	[46]

Notes: (a) The habitat details or bioactivity have not been disclosed yet.

**Table 2:** Comparative predictive drug-likeness parameters of cyclic glycine-proline using empirical rule parameters.

ADME Parameters ^a^	cGP	“Rule of Two” [79]	Lipinski’s Rule [81]	Veber’s Rule [82]	Clark&Lobell’s Rule [85,86]
MW	154.2	<200	<500	<500	<450
cLogP	−1.37/−0.29 ^c^	<2	≤5	--	1–3
H Don	2	≤2	≤5	Sum ≤ 10	--
Hacc	1	≤4	≤10		<6
Rot B	0	≤4	--	≤10	--
Fsp_3_	0.73	0.2~1	--	--	--
TPSA ^b^	49.4 Å^2^	--	--	<140 Å^2^	<60–70 Å^2^

Note: (a) The data were collected from the SwissADME website [75]; (b) TPSA was calculated using Molinspiration free services [76]; (c) Indicates data retrieved from SciFinder. MW, molecular weight; cLogP, calculated logarithm of the partition coefficient P between lipid and water; H Don, the number of hydrogen bond donors; Hacc, the number of hydrogen bond acceptors; RotB, the number of rotatable bonds; Fsp3, the ratio of saturated carbon atoms; TPSA, the total polar surface area of the molecule.

## Data Availability

All data in this article is openly available without any restrictions.
